# Systematic review of community participation interventions to improve maternal health outcomes in rural South Asia

**DOI:** 10.1186/s12884-018-1964-1

**Published:** 2018-08-10

**Authors:** Binod Bindu Sharma, Lisa Jones, Deborah Joanne Loxton, Debbie Booth, Roger Smith

**Affiliations:** 1 Mothers and Babies Research Centre, Hunter Medical Research Institute, Lookout Rd, New Lambton Heights, NSW 2305 Australia; 2 The University of Newcastle, Callaghan, Australia; 3 Department of Neonatology, John Hunter Hospital, Lookout Rd, New Lambton Heights, NSW 2305 Australia; 4 Department of Obstetrics and Gynaecology, Sydney Medical School, University of Sydney, Camperdown, Australia; 5 Priority Research Centre for Generational Health and Ageing, The University of Newcastle, Callaghan, Australia; 6 Hunter Medical Research Institute, Lot 1 Kookaburra Circuit, New Lambton Heights, NSW 2305 Australia; 7 Mothers and Babies Research Centre, Hunter Medical Research Institute, Lot 1 Kookaburra Circuit, New Lambton Heights, NSW 2305 Australia; 8 Department of Endocrinology, John Hunter Hospital, Lookout Rd, New Lambton Heights, NSW 2305 Australia; 9 Priority Research Centre for Reproductive Science, The University of Newcastle, Callaghan, Australia

**Keywords:** Community, Networks, Rural, Pregnancy, Antenatal care, Delivery, Pregnancy complications, Maternal death

## Abstract

**Background:**

This is a systematic review on the effectiveness of community interventions in improving maternal health care outcomes in South Asia.

**Methods:**

We searched electronic databases to June 2017. Randomised or cluster randomised studies in communities within rural/remote areas of Nepal, Bangladesh, India and Pakistan were included. Data were analysed as risk ratios (RR) or odds ratios (OR), and effects were adjusted for clustering. Meta-analyses were performed using random-effects and evidence quality was assessed.

**Results:**

Eleven randomised trials were included from 5440 citations. Meta-analysis of all community interventions combined compared with control showed a small improvement in the number of women attending at least one antenatal care visit (RR 1.19, 95% CI 1.06 to 1.33). Two community mobilisation sub groups: home care using both male and female mobilisers, and education by community mobilisers, improved the number of women attending at least one antenatal visit. There was no difference in the number of women attending at least one antenatal visit for any other subgroup. There was no difference in the number of women attending 3 or more antenatal visits for all community interventions combined, or any community subgroup. Likewise, there was no difference in attendance at birth between all community interventions combined and control. Health care facility births were modestly increased in women’s education groups (adjusted RR (1.15, 95% CI 1.11 to 1.20; 2 studies)). Risk of maternal deaths after 2 years (RR 0.63, 95% CI 0.24 to 1.64; 5 studies), and 3 years (RR 1.11, 95% CI 0.52 to 2.36; 2 studies), were no different between women’s education groups and control. Community level mobilisation rather than health care messages at district level improved the numbers of women giving birth at health care facilities (RR1.09 (95%CI 1.06 to 1.13; 1 study)). Maternal health care knowledge scores improved in two community-based interventions, one involving education of male community members.

**Conclusion:**

Women’s education interventions may improve the number of women seeking birth at a health care facility, but the evidence is of low quality. No impact on maternal mortality was observed Future research should explore the effectiveness of including male mobilisers.

**Trial registration:**

This systematic review is registered with PROSPERO CRD42016033201.

**Electronic supplementary material:**

The online version of this article (10.1186/s12884-018-1964-1) contains supplementary material, which is available to authorized users.

## Background

More than 800 women worldwide die each day from pregnancy and childbirth-related complications [1] with 99% of these occurring in low and lower middle-income countries [2–5]. South Asian and sub-Saharan African countries bear the highest burden of maternal death [6]. The widest level of disparity in maternal mortality is between low/lower middle, and high-income countries. The lifetime risk of maternal death in high income countries is one in 3700, compared with one in 160 live births in low income countries [7]. It has been estimated that 16% to 33% of all maternal mortality may be avoided by preventing complications through the provision of skilled personnel at birth [8].

Quality care throughout pregnancy and childbirth is associated with good maternal and infant outcomes [9]. Antenatal care uptake in rural settings is dependent on social and cultural factors [10]. Increased access to antenatal care, provision of skilled birth attendants and pregnancy care awareness programs at the local level contribute to safer pregnancies and childbirth [11]. In rural families, money, food and other logistics have all been seen to have an important influence on maternal health outcomes [12]. For instance, in Nepal, pregnancy and childbirth are considered to be the domain of women [13]. Although mothers-in-law commonly make decisions on pregnancy-related issues [14], permission must be sought from the male head of the household for any costs associated with seeking pregnancy care. As it is very uncommon for men to take an interest in pregnancy-related care issues, the resulting lack of communication limits women’s access to pregnancy care [15]. Gender disparity and discrimination is common in South Asian countries like Nepal and is particularly prevalent in rural settings [16].

Health care interventions are often limited to a rigid and structured operational framework rather than being designed to meet the socio-cultural and economic realities of the communities they serve [17]. National programs and strategies often fail to consider the hardship imposed by distance and lack of infrastructure that is peculiar to people living within rural settings [18]. Poor social status among women in South Asian countries is a great contributor to lack of family planning and a rising population growth. This “feminisation of poverty” in the region is a fundamental anomaly that has impaired societal development [19]. Engaging local people to educate and mobilise the community has the capacity to provide multidimensional benefits [20] such as: helping to modify practices [21], encouraging a sense of community [22], and emboldening the identification of local methods to address problems [23].

Changing people’s attitudes is required, but the best method for doing this is unclear. This systematic review aimed to compare the overall effect of different approaches to community participation in maternal health care education compared with health service or control/standard care interventions, on important maternal health outcomes. Furthermore, this systematic review aimed to examine which interventions promote husband, family and community awareness and involvement in maternal health care and result in better maternal health care-seeking and utilisation of maternal health services for improved maternal health outcomes in rural South Asian countries.

The overall objective of this systematic review was to compare the effectiveness of interventions to promote family and community participation in maternal health care against standard health care and health service led programs on the outcomes: indicators of maternal health care knowledge improvement, maternal health care utilisation (antenatal care, facility birth, skilled birth attendant use), and maternal mortality in rural-remote regions of the South Asian countries, Bangladesh, India, Nepal, and Pakistan.

## Methods

### Search strategy and selection criteria

Database searches were initially conducted in November 2015 and updated in June 2017. No restriction was placed on language or year of publication (refer Medline search strategy, Additional file 1). Hand searches were conducted on the reference lists of included studies.

This systematic review and meta-analysis were conducted according to a prospectively registered protocol (PROSPERO CRD42016033201) dated 14 January, 2016 and reported as per the Preferred Reporting in Systematic Reviews and Meta analyses (PRISMA) Guidelines [24].

We included cluster randomised trials or randomised trials evaluating different community health promotion interventions compared against each other, or against control or health service-based interventions, involving women, men, family and community members living within rural areas of Nepal, Bangladesh, India and Pakistan. We excluded studies of urban populations, and middle to high-income countries. Primary outcomes included: indicators of improved knowledge among women of childbearing age, their husbands/partners, family and community members, skilled provider attendance at birth (formal provider, traditional birth assistant), delivery at a health care facility and maternal mortality. The secondary outcome was male involvement in supporting access to maternal health care provision defined as; the supportive role played by men particularly husband, father-in-law and others with decision making capacity to access care during pregnancy, childbirth and postnatal period [25]. Traditional birth assistant (TBA) is defined as; *“a person normally a female, who assists mothers during childbirth and who initially learns her skills delivering babies by herself or by working with another more experienced TBA”* [26].

Two authors (BBS, LJ) independently extracted data from the full-text articles using the Covidence program [27]. Any differences in opinion were resolved by consensus. Data were cross-checked for accuracy by both authors. Reviewers (BBS, LJ) independently assessed the risk of bias (low, high, or unclear) (refer Additional file 2) of all included trials using the Cochrane ‘Risk of bias’ tool and evidence quality was assessed using GRADE [28, 29]. Any disagreements were resolved by discussion until consensus was reached. Data were analysed using Review Manager Version 5.3. Meta-analyses were performed using fixed-effects modelling, random-effects modelling was substituted in situations where heterogeneity (I^2^) was greater than 20%. Publication bias was assessed by visual inspection of funnel plots and incorporated in GRADE quality assessment [30]. Results for dichotomous data were presented as risk ratios (RR) with 95% confidence intervals (CI) [30. Continuous data were reported using mean difference (MD) or standardised mean difference (SMD) where appropriate [30]. All trials randomised the intervention at village or district level. To avoid unit of analysis error caused by clustering, the intra-cluster coefficient (ICC) was used to calculate the effective sample size of both intervention and control arms which was then used to adjust the standard error for all analyses [30]. Where not provided in the study, ICC estimates were imputed from other sources [31]. Data were entered into the analysis as inverse ratios (logOR or logRR) and adjusted standard errors. Sensitivity analyses were conducted including only trials of moderate quality or above [30].

In order to consider a study as overall having low risk of bias we defined that it had to have none of the domains considered as high risk of bias and at least four (not counting ‘Other biases’) considered as low risk of bias, and two of these must include ‘random sequence generation’ and ‘incomplete outcome data’ as the domains most likely to influence overall measurements of effect.

## Results

**Fig. 1:**
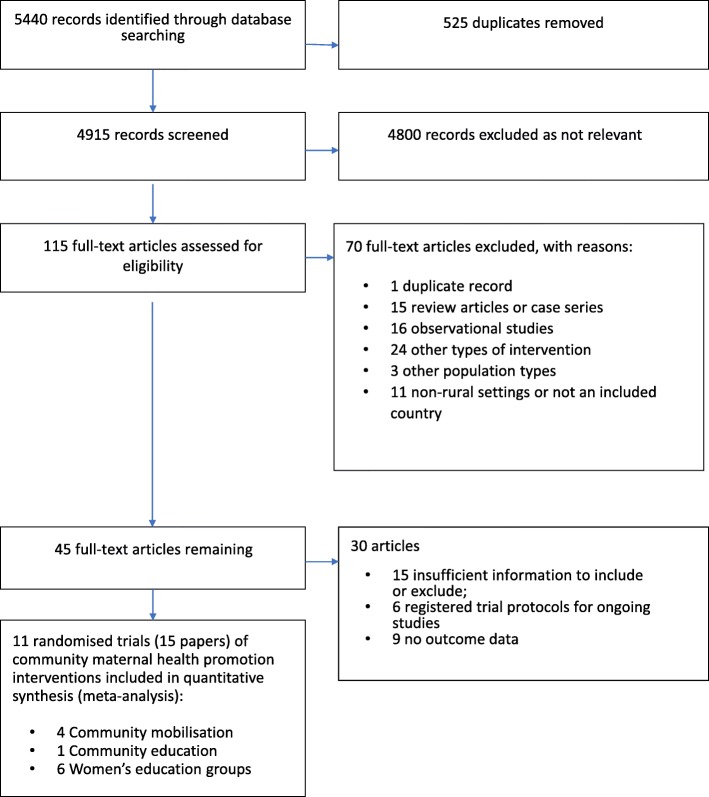
Study selection

We identified 5440 citations in our search strategy, removed 525 duplicates and screened title and abstracts of 4915 (refer to Fig. 1 ‘Study selection’), resulting in 115 articles which we assessed in full text against the inclusion and exclusion criteria. Of the 45 articles remaining, we excluded nine studies that did not report any of the stated review outcomes, 15 contained insufficient information to make a judgement on whether they should be included or excluded, and six related to registered trial protocols awaiting publication of results. Of the 15 remaining publications there were only 11 independent studies as four studies had appeared in more than one publication.

Pregnant women and women of reproductive age (15 to 49 years or < 50 years) were most commonly targeted (9 studies), in addition to their in-laws (1 study), community members of any age (2 studies), and both women and men of reproductive age (1 study) (Tables 1 and 2). All studies were from rural and remote areas of low or lower middle income South Asian countries sharing a similar burden of maternal morbidity and mortality, including India (3 studies), Nepal (2 studies), Bangladesh (4 studies) and Pakistan (2 studies). Population sample sizes ranged from 1058 to 29,889 pregnant women, communities (health units, rural unions) or live births.

**Table 1:** Characteristics of studies. Community interventions versus health services, standard care or control

Study/Year published Country Duration of intervention	Design	Population	n	Risk of Bias ^a^	Intervention	Control	Outcome	OR		RR	
Azad [42] Bangladesh 2005 to 2007	Cluster RCT 18 clusters	Women aged 15 to 49 years having given birth during the study period	29,889	1. Low 2. Unclear 3. Unclear 4. Low 5.High 6.Low 7.Low Overall: High	Women’s education groups plus health system improvement	Control (health system improvement)	Antenatal care				
							any	0.78 [0.51, 1.19]*		0.91 [0.76, 1.09]**	
							≥ 4 visits	0.15 [0.06, 0.40]*		0·79 [0.46, 1.37]***	
							Skilled birth attendant formal provider	0.45 [0.19, 1.11]*		0.90 [0.72, 1.14]**	
							Delivery at a health care facility	0.75 [0.62, 0.89]***		0.97 [0.77, 1.24]**	
							Maternal deaths				
							2 years	–		1.80 [1.2, 3.17]*	
							3 years (N = number of live births for all outcomes above)	–		1.67 [1.00, 2.79]*	
							MMR (over 3 years) (live births per 100,000)	388.9 vs. 189.10		2.02 [1.11, 3.68]**	
										1.74 [0.97, 3.13]#	
Baqui [49] Bangladesh 2003 to 2006	Cluster RCT 24 clusters	All married women of reproductive age (15–49 years)	5110	1.Low 2.Low 3.Unclear 4.Low 5.High 6.Low 7.Low Overall: High	Community mobilisation: home care by female mobilisers visiting every 8 months	control	Antenatal care Any (N = number of live births)	1.70 [1.07, 2.68]*		1.13 [0.93, 1.36]*	
					Community mobilisation: Community care by male mobilisers visiting every 10 months and female mobilisers visiting every 4 months	control	Antenatal care				
							Any (N = number of live births)	2.67 [1.70, 4.21]*		1.47 [1.21, 1.70]*	
					Community mobilisation: Both community and home care	control	Antenatal care				
							Any (N = number of live births)	2.13 [1.33, 3.39]*		1.37 [1.15, 1.63]*	
Bhutta [50] Pakistan 2006 to 2008	Cluster RCT 16 clusters	Pregnant women and women of reproductive age (15–49 years)	4474	1.Low 2.Unclear 3.Unclear 4.Low 5.Low 6.Low 7.Low Overall: Low	Community mobilisation	Healthcare workers (standard healthcare)	Antenatal care				
							Any	1.64 [1.03, 2.62]*		1.20 [1.01, 1.42]*	
							≥ 4 visits	1.51 [0.79, 2.88]*		1.44 [0.75, 2.77]*	
							Delivery at a health care facility (any) (N = number of women for all outcomes)	1.53 [1.36, 1.72]***		1.24 [1.17, 1.32]***	
Darmstadt [51] Bangladesh 2004 to 2006	Cluster randomised trial 12 clusters	All married women of reproductive age (15–49 years)	3491	1.Low 2.Unclear 3.Unclear 4.Low 5.Low 6.Low 7.Low Overall: Low	Community education by community health care workers	Control	Antenatal care				
							Any	2.29 [1.34, 3.91]*		1.40 [1.12, 1.75]*	
							Skilled birth attendant	Not reported		Not reported	
							Delivery at health care facility (N = number of women for all outcomes)	1.20 [0.62, 2.29]***		1.19 [0.63, 2.27]***	
							Maternal danger sign knowledge score:	Mean score Intervention		Mean score Control	
								Pre	Post	Pre	Post
							a. Antenatal [0–10]	1.0	2.9	1.1	2.2
							b. Labor/delivery [0–11]	1.1	2.4	1.2	1.9
							c. Postpartum [0–9]	1.0	2.5	1.0	2.5
Fottrell [52] Bangladesh 2005 to 2007	Cluster randomised trial 18 Clusters	Ever-married women of reproductive age (15–49 years) who were permanent residents including in-laws and adolescent girls	17,940	1.Low 2.Unclear 3.Unclear 4.Low 5.Low 6.Low 7.Unclear Overall: Low	Women’s education groups plus health system improvement	Control (health system improvement)	Antenatal care				
							≥ 4 visits	1.37 [0.99–1.88]**		1.28 [0.13, 12.11]*	
							Skilled birth attendant	0.54 [0.38, 0.78]**		0.92 [0.71, 1.20]*	
							Delivery at health care facility (any) (N = number of births for above outcomes)	1.05 [0.88, 1.25]**		0.94 [0.55, 1.59]***	
							Maternal deaths 2 years (N = number of live births)	–		0.59 [0.30, 1.18]*	
							MMR (over 2 years) (per 100,000 live births)	153.4 vs. 276.10		0.74 [0.34, 1.64]**	
Midhet** [53] Pakistan 1998–2002	Cluster randomised trial 24 clusters	Women and men of reproductive age (all ever-married women under 50 years of age)	2564	1.Low 2.Unclear 3.Unclear 4.Low 5.High 6.Unclear 7.Low Overall: High	Women’s and men’s education groups	Control	Antenatal care				
							Any	2.83 [1.60, 5.00]**		1.35 [0.81, 2.25]*	
							Delivery at health care facility (any) (N = number of pregnant women for all outcomes)	1.3 [0.6, 2.7]**		1.28 [0.84, 1.96]***	
					Women’s education groups	Control	Antenatal care				
							Any	2.45 [1.40, 4.30]**		1.32 [0.79, 2.20]*	
							Delivery at a health care facility (N = number of pregnant women for all outcomes)	1.3 [0.7, 2.5]**		1.32 [0.86, 2.02]***	
					Both interventions combined (women’s education groups and women’s and men’s education groups)	Control	Antenatal care				
							Any	1.38 [0.82, 1.34]*		1.33 [0.84, 2.10]*	
							Delivery at health care facility (any) (N = number of pregnant women for all outcomes)	1.46 [0.99, 2.15]***		1.43 [0.99, 2.07]***	
Osrin [54] Nepal 1998–2000	Randomised trial 24 Clusters	Women (aged 15–49 years) and key members of the community in improving perinatal health outcomes	4241	1.Low 2.Unclear 3.Unclear 4.Low 5.Low 6.Low 7.Low Overall: Low	Women’s education groups	Control	Antenatal care				
							Any	2.82 [1.41, 5.62]**		1.32 [1.08, 1.60]*	
							(N = number of pregnancies) Skilled birth attendant				
							Any	2.50 [1.51, 4.16]*		2.26 [1.43, 3.57]*	
							formal health provider (doctor or nurse)	3.13 [1.62, 6.03]**		2.96 [1.50, 5.84]*	
							Traditional birth assistant (N = number of deliveries)	1.70 [0.93, 3.11]**		1.71 [0.89, 3.31]*	
							Delivery at health care facility	3.54 [1.56, 8.05]**		3.38 [2.57, 4.45]***	
							Maternal deaths 2 years			0.18 [0.14, 0.24]*	
							MMR (over 3 years) (per 100,000 live births)	69 vs 341		0.22 [0.05, 0.90]**	
Sharma 2018 [34] Nepal	Pre and post-test randomised	All community members (all ages eligible)	1572	1.Low 2.Unclear 3.Unclear 4.Low 5.Low 6.Low 7.Unclear Overall: Low	Community mobilisation: Community singing to deliver healthcare messages	Control	1. Mean change in knowledge scores (se):				
							a. Importance of antenatal examination (out of 7)	2·12 [0·06] vs 4.89 [0.06]			
							b. Importance of supplementary diet and rest during pregnancy (out of 9)	3·71 [0·07] vs 6·84 [0·06]		0.12 [−0.22, 0.46]	
							c. Importance of delivery care (out of 12)	2·95 [0·08] vs 5·09 [0·07]			
							d. Importance of childbirth planning (out of 8)	2·81 [0·08] vs 5·50 [0·06]		−0.71[−1.3, −0.11]	
							e. Overall knowledge (out of 36)	11·60 [0·24] vs 22·33 [0·18]		−1.02 [−2, −0.03]	
Tripathy 2010 [55] India 2005 to 2008	Cluster RCT 18 clusters	Women of reproductive age (15–49 years)	17,335	1.Low 2.Unclear 3.Unclear 4.Low 5.Unclear 6.Low 7.Unclear Overall: Unclear	Women’s education groups plus health system improvement	Control (health system improvement)	Antenatal care				
							Any	0.97 [0.48, 1.97]**		1.11 [0.99, 1.23]*	
								1·60 (0·65–3·92)#			
							> 3 visits	0.68 [0.37, 1.24]**		0.70 [0.57, 0.87] *	
							Skilled birth attendant				
							Any	0.52 [0.37, 0.74]**		0.70 [0.57, 0.87]*	
							Formal provider	0.59 [0.37, 0.94]*		0.67 [0.46, 1.00]*	
							Traditional birth assistant	0.82 [0.43, 1.56]*		0.88 [0.68, 1.13]*	
							Delivery at health care facility(any) (*N* = number of pregnant women for all outcomes)	0.64 [0.39, 1.04]**		0.71 [0.66, 0.75]***	
							Maternal deaths				
							2 years			0.82 [0.51, 1.33]*	
							3 years			0.77 [0.53, 1.13]*	
							MMR (over 3 years) (per 100,000 live births)	517.5 vs 680.3		0.70 [0.46, 1.07]**	
Tripathy 2016 [56] India 2009 to 2012	Randomised Controlled Trial 30 clusters	Women of reproductive age (15–49 years)	7100	1.Low 2.Unclear 3.Unclear 4.Low 5.Unclear 6.Low 7.Low Overall: Unclear	Women’s education groups plus health system improvement	Control (health system improvement)	Antenatal Care				
							Any	0.82 [0.35, 1.92]**		0.90 [0.75, 1.07]*	
								0·63 [0·35–1·16]#		1.17 [0.78, 1.77]*	
							> = 3 visits (N = number of births)	1.08 [0.58, 2.01]*		1.16 [1.12, 1.20]**	
							Delivery at health care facility	1.23 [0.58, 2.60]**		0.63 [0.25, 1.42]**	
							Maternal deaths 2 years (N = number of live births for all of the above outcomes)	–		0.63 [0.26, 1.55]*	
							MMR (over 2 years) (per 100,000 live births)	222 vs. 349 0.63 [0.25, 1.42]***			

^a^Risk of Bias tool (Cochrane)

^*^Adjusted estimate using outcome specific ICC

^**^Adjusted estimate reported by authors – adjusted for clustering and stratification

^***^Unadjusted estimate reported by authors

^#^Adjusted estimate reported by authors – adjusted for clustering, stratification and baseline covariates

**Table 2:** Community participation interventions versus combined health service and community interventions or other types of community-based interventions

Study/Year published Country Duration of intervention	Design	Population	n	Risk of Bias ^a^	Community and Health Service Intervention	Health Service Intervention	Outcome	OR	RR
Acharya 2015 [57] India 2005 to 2011	RCT	Women becoming pregnant or giving birth during the study period (average age 27 years) and their families		1. Low 2. Unclear 3. Unclear 4. Low 5. Low 6. Low 7. Unclear Overall: Low	Community mobilisation at community level (L2)	Community mobilisation at community level combined with health care messages at district level (L1 and L2)	Antenatal care		
							Any	1.21 [0.86, 1.70]*	1.04 [0.97, 1.10]*
							Skilled birth attendant	0.90 [0.56, 1.43]*	0.91 [0.61, 1.35]*
							Delivery at a health facility (*N* = number of women for all outcomes)	1.10 [1.03, 1.17]**	1.04 [1.02, 1.07]**
					Community mobilisation at community level (L2)	Healthcare messages at district level (L1)	Antenatal care:		
							Any		1.07 [0.98, 1.17]*
							Skilled birth attendant		0.83 [0.64, 1.07]*
							Delivery at a health facility		1.09 [1.06, 1.13]**
							(*N* = number of women for all outcomes)		
					Community mobilisation at community level (L2) involving ‘Sure Start’ community field workers working directly with ASHAs and strengthening village health and sanitation committees, and health care messages at district level (L1)	Healthcare messages at district level (L1)	Antenatal care:		
							Any		0.76 [0.19, 3.08]*
							Skilled birth attendant		0.83 [0.64, 1.07]*
							Delivery at a health facility (any)		1.05 [1.02, 1.08]**
							(*N* = number of women for all outcomes)		
Baqui [49] Bangladesh 2003 to 2006	Cluster RCT 24 clusters	All married women of reproductive age (15–49 years)		1.Low 2.Low 3.High 4.High 5.High 6.Low 7.Low Overall: High	Community mobilisation: home care with female mobilisers visiting every 8 months	Community mobilisation: Community care with Male mobilisers visiting every 10 months and Female mobilisers visiting every 4 months	Antenatal care:		
							Any		0.76 [0.19, 3.08]*
							(N = number of live births, % = cluster averages)		0.94 [0.89, 1.00]**
Midhet** [53] Pakistan 1998–2002	Cluster randomised trial 32 clusters	Women and men of reproductive age (all ever-married women under 50 years of age)		1.Low 2.Unclear 3.Unclear 4.Unclear 5.High 6.Unclear 7.Low Overall: High	Women’s and men’s education groups	Women’s education groups	Antenatal care		
							Any	1.42 [0.99, 2.05]*	1.05 [0.89, 1.24]*
							Delivery at a health facility (*N* = number of pregnant women for all outcomes)	1.01 [0.65, 1.56]**	1.01 [0.67, 1.53]**

^a^Risk of Bias tool (Cochrane)

*Adjusted estimate using outcome specific ICC (Page 1)

**Adjusted estimate reported by authors – adjusted for clustering and stratification

***Unadjusted estimate reported by authors

^#^Adjusted estimate reported by authors – adjusted for clustering, stratification and baseline covariates

Eleven studies randomly allocated communities to community interventions; 10 studies compared a community intervention to a standard health care or control group (Table 1), and one (Acharya) compared different types of community intervention combinations at village or district level (Table 2). Two studies (Baqui, Midhet) assessed a community intervention against standard care or control group and provided data for comparisons between different community interventions. Four studies (Acharya, Baqui, Bhutta, Sharma) involved the use of community members to mobilise their community to take part in maternal health education and take practical steps to improve maternal health care within the community. Baqui compared the use of both male and female community mobilisers with the use of female mobilisers only. Darmstadt used community health workers to deliver a community education intervention. Six studies (list studies) involved the participation of community women in maternal health education groups. The intervention periods occurred over approximately 2 years.

In addition to examining the results for each outcome, all community interventions were compared against:

- standard care or control (10 studies) (Table 1), and;
- other community interventions (3 studies) (Table 2);

We identified the following post-hoc Community intervention subgroups:

1. Use of community mobilisers to deliver maternal health education;
  - Home care by male and female mobilisers.
  - Community care by female mobilisers.
2. Community education by health care workers.
3. Women’s maternal health care education groups.
4. Women’s and men’s maternal health care education groups.

As the design of cluster randomised studies is likely to have made it difficult to provide blinded participation and assessment, we rated all studies not blinded or not reporting blinding, as unclear risk performance and detection bias. The main domains of interest for these study designs were random sequence generation, allocation concealment, incomplete outcome data and subjective reporting.

Overall, six studies were assessed as low risk of bias, two (Tripathy 2010, 2016) as unclear risk, and three (Azad, Baqui, Midhet) as high risk. Azad, Baqui and Midhet reported > 10% losses of population outcome data to the final analysis over the study follow-up period. Although population outcome data losses at follow-up were < 10%, Tripathy 2010 excluded twice as many women and live births in the control group (169 women, 171 births from a total of 9260 births; 1.8% losses) compared with that of the intervention group (83 women, 84 births from a total of 9770 births; 0.9%) due to deaths, stillbirths and migration. Similarly, Tripathy 2016 excluded a greater percentage of population outcome data from the final analysis in the intervention group (6238 women from 82,702; 7.6%) compared with the control group (28 women from 73,817; 0.04%).

The individual results of all included studies are shown in Tables 1 and 2.

Two studies (Darmstadt, Sharma) reported improved mean knowledge scores among women of childbearing age, their partners, and family and community members. There was a small improvement in knowledge of maternal danger signs RR 1.40 (95% CI 1.12, 1.75) in receiving community education intervention by community health workers compared with a control group, whilst Sharma demonstrated improvement in maternal health care knowledge among community members receiving health care messages in a community singing intervention compared with control. Post-intervention, knowledge doubled in the intervention group from a mean of 11.60/36 to 22.33/36, an increase of 10.69 points [*P* < 0.001], with only a modest change in the control population [17.48/36 to 18.26/36].

**Fig. 2:**
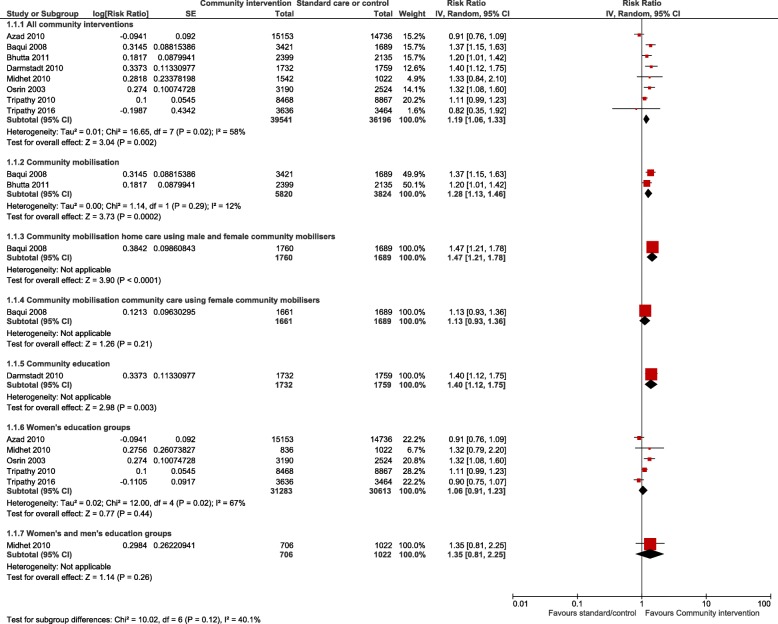
Forest plot of comparison: 4 Community interventions vs. standard health care, control or no intervention (all adjusted RR), outcome: Antenatal Care (any) adjusted RR

**Fig. 3:**
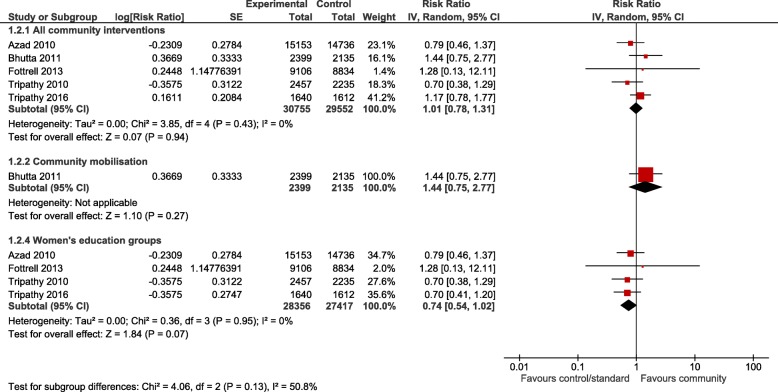
Forest plot of comparison: 1 Community interventions vs. standard health care, control or no intervention (all adjusted RR), outcome: Antenatal Care (≥ 3 visits)

**Fig. 4:**
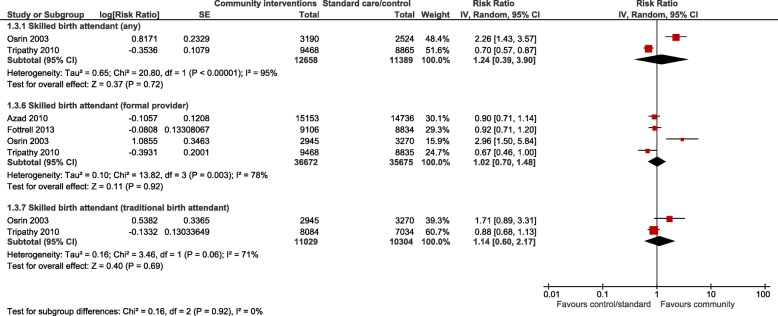
Forest plot of comparison: 1 Community interventions vs. standard health care, control or no intervention (all adjusted RR), outcome: Skilled birth attendant

**Fig. 5:**
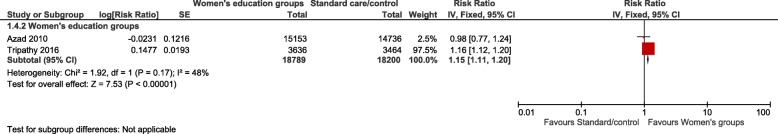
Forest plot of comparison: 1 Community interventions vs. standard health care, control or no intervention (all adjusted RR), outcome: Delivery at a health facility (all adjusted RR)

**Fig. 6:**
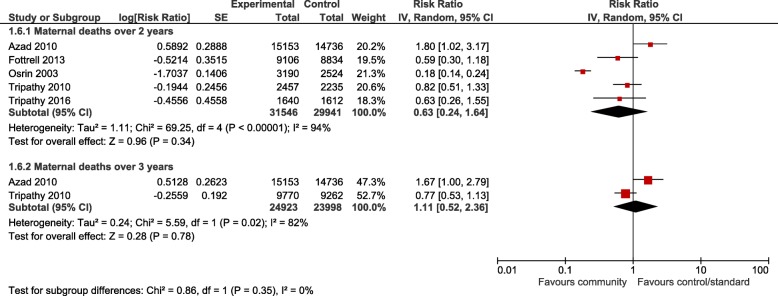
Forest plot of comparison: 1 Community interventions vs. standard health care, control or no intervention (all adjusted RR), outcome: Maternal deaths (N = number of live births) STATA one-way ICC

For the meta-analyses, all community interventions combined increased attendance of at least one antenatal visit compared with control by an average of 19% (RR 1.19, 95% CI 1.06 to 1.33; participants = 75,737; studies = 8; I^2^ = 58%) (Fig. 2). Intervention sub groups: use of community mobilisers, community care using female mobilisers, home care using both male and female mobilisers, and community education by health workers, had a similar effect (Fig. 2). However, there was no difference in effect in the number of women seeking at least one antenatal visit for community care using female mobilisers, women’s education groups, and women’s and men’s education groups. There was no difference in the number of women attending three or more antenatal visits from any community interventions, or any intervention subgroups, compared with control (Fig. 3). Likewise, there was no difference in attendance by a person of any skill level, formal provider (doctor or nurse) or traditional birth attendant between community intervention and control groups (Fig. 4). Deliveries in health facilities were increased in women’s education groups for meta-analyses of studies reporting adjusted RR 1.15 (95% CI 1.11 to 1.20; participants = 36,989; studies = 2; I^2^ = 48%) but not in meta-analyses of studies reporting adjusted OR 1.19 (95% CI 0.71 to 1.99; participants = 49,590; studies = 4; I^2^ = 76%) (Fig. 5). Estimates of RR and OR are more likely to be similar when the number of events is rare, which may explain the difference between OR and RR for antenatal care attendance as the number of events are comparatively much higher. For this reason, we have based our discussion and conclusions on RR in preference to OR. There was no difference in risk of maternal deaths at two (RR 0.63, 95% CI 0.24 to 1.64; participants = 61,487; studies = 5; 94%), and 3 years (RR 1.11, 95% CI 0.52 to 2.36; participants = 48,921; studies = 2; I^2^ = 82%), between women’s education groups and health service, standard care or control (Fig. 6).

No studies reported on the secondary outcome, indicators of male involvement (fathers-in-law and male partners) in maternal health care.

In addition to the varying results and evidence quality in the interventions and intervention subgroups described above, further investigation of potential contributors to heterogeneity by population (country), and outcome measurement (follow-up period 2 years vs. 3 years) subgroups showed no reduction in heterogeneity for any combination of community mobilisation interventions versus standard healthcare or control. There were insufficient data to explore the effect of these on the remaining comparisons.

## Discussion

Overall, meta-analysis of all community interventions combined showed modest benefits of in terms of improving attendance of at least one antenatal care visit but not the recommended four antenatal visits recommended by the World Health Organisation. Meta-analysis showed no difference in assistance of birth attendants at birth between all community interventions combined, or any community intervention subgroup and control. Women’s education group interventions rather than health service or control, and community level mobilisation rather than health care messages at a district level, increased the numbers of women delivering at a health care facility. There was no difference in maternal mortality for meta-analyses between community intervention groups, intervention subgroups and health service, standard care or control. A community-based health promotion intervention [32], including men, women and community members from rural Nepal, showed an improvement in overall knowledge scores for antenatal care and skilled birth attendance.

There were several limitations to the findings of this review. Evidence quality varied across the outcomes for the three main comparisons (refer Additional file 2 GRADE tables). There was moderate to considerable heterogeneity for all outcomes. As the majority of studies were primarily designed to evaluate neonatal rather than maternal outcomes, there were no outcome specific ICC available to calculate adjusted estimates of effect for some maternal outcomes, meaning that some study data for some outcomes, such as institutional delivery, could not be included in the analysis. Studies reported different maternal health knowledge outcome categories which could not be combined in a meta-analysis.

Our review demonstrated improved maternal health outcomes where male community mobilisers were involved in home-based community interventions. Previous evidence suggests that the need to prioritise male involvement in maternal health care education in addition to measures that aim to improve women’s education and their status in the family [33]. Involvement of the male family members in maternal health care education is of particular importance in low socioeconomic and uneducated community environments [34]. Intervention strategies involving men and community leaders in maternal health care programs in Bolivia resulted in improved maternal health outcomes in a low resource environment [35]. A study carried out in Maharashtra, India concluded that the maternal mortality ratio was three times higher among women with uneducated husbands compared to the group of women with college-educated husbands [36]. The involvement of husbands in the utilization of maternal care needs to be included as equally important as the improvement of women’s education and their status in the family [33]. Although the involvement of male (fathers-in-law and husbands) members in maternal health care is critical, [37] only one study reported this outcome.

Previous research has concluded that women from deprived communities with poor access to health care and low levels of education have an increased risk of mortality [38]. All community interventions, and use of community mobilisers were more effective than health service, standard care or control, while women’s education groups, female mobilisers, women’s and men’s (couples) education were not. The selection of suitable interventions is critical. This could be one of the reasons that, although worldwide maternal mortality seems to be decreasing, there has not been a similar change for women in rural/remote areas of lower and lower-middle income countries in South Asia [39]. A study carried out in Tamil Nadu, India showed improved maternal health care knowledge among both males and females following education via mobile phone text messaging. Ninety eight percent of participants surveyed responded that text messaging was an effective means of health education [40]. Although this study was carried out in a rural setting, the high level of literacy in Tamil Nadu may have influenced this result. This evidence may therefore not apply to all rural environments of South Asian countries, as use of mobile phone demands many prerequisites such as: ability to read and write, buying a mobile phone, and accessibility to mobile networks in remote locations.

Our meta-analysis found that there was no difference in the risk of maternal death in communities allocated to women’s education groups compared to standard care or control. In a systematic review of women’s participatory groups in Nepal, Malawi, India and Bangladesh, also no difference in overall maternal mortality was found [41]. After having further divided the women’s group according to the percentage of pregnant women attending, Prost [41] found that maternal mortality was halved only in those groups having > 30% pregnant women (OR 0.51, 95CI 0.29–0.89). Similarly, in our analysis, the only study showing increased risk of mortality, Azad [42], at 3 %, had the lowest proportion of pregnant women attending women’s education groups out of all the studies in the meta-analysis. It is possible that women’s education interventions may need to include a larger proportion of pregnant women in order to be more successful in reducing maternal mortality.

Another systematic review assessing the impact of community interventions on maternal health in resource poor economies, revealed that community-based programs integrated with multiple interventions greatly improved maternal health outcomes [43]. A review of randomised trials aiming to improve antenatal care practice demonstrated a reduction in maternal mortality (OR 0.62, 95% CI 0.39–0.98) [44]. The effectiveness of these community level interventions on maternal outcomes challenges the viewpoint that these programs are not worth the cost.

Women’s education group interventions did not improve the use of skilled care at birth. It is possible that the involvement of men who control finances and family decisions and improving infrastructure may have improved this outcome. For pregnant women to obtain necessary antenatal visits and skilled care during childbirth, it is necessary to discuss and plan pregnancy care with the men who are responsible for decision-making within the family [45]. Antenatal care is essential to help prevent pregnancy complications and minimize maternal mortality.

Our meta-analysis showed that the numbers of women attending at least one antenatal visit were greater among women receiving any type of community intervention and intervention sub groups. This finding is supported by a study conducted in Nepal that demonstrated improvement in maternal health care outcomes in rural communities using a female facilitator in organizing monthly meetings with women’s groups [46].

Increased access to antenatal care, provision of skilled birth attendants and pregnancy care awareness programs at local level contributes to safer pregnancies and childbirth [11]. Previous studies have concluded that delivery in a health care facility offers much needed emotional support to pregnant women [47]. Presence of skilled professionals, lifesaving drugs and equipment help to reduce the risk of complications and death of mother and baby [48]. However, our meta-analysis demonstrated unexpected outcomes of community intervention. For example, none of the interventions were effective in increasing the number of women receiving any level of trained or skilled assistance at birth.

## Conclusion

A range of community interventions are likely to be successful in improving antenatal care attendance. Pregnant women receiving women’s education group interventions were more likely to deliver at a health care facility. However, women’s education groups were less likely to seek antenatal care or have a formal provider attend at birth. The contributing factors to this are unclear, but it has been previously suggested [41] that the varying proportion of pregnant women attending women’s education interventions among the included studies may have been an important factor. Moderate quality evidence from a single study suggests including male alongside female mobilisers in community mobilisation home care programs [34] may improve the success of women’s groups on maternal health care outcomes. Further research is needed on the impact of male involvement in community interventions to supplement women’s involvement in community mobilisation, which up until now has been the primary focus, and the impact of couple’s education in preference to women only. National health guidelines should include evidence from current systematic reviews of randomised trials, when planning interventions to promote community education on maternal health care.

## Additional files

Additional file 1: The Medline search strategy. (DOCX 19 kb)

Additional file 2: Title of data: GRADE tables. List of outcomes, relative effects (95% CI), number of participants (studies) and certainty of the evidence (GRADE). (PDF 73 kb)

## Abbreviations

CI: Confidence interval
GRADE: Grading of Recommendations Assessment, Development and Evaluation.
ICC: Interclass correlation coefficient
MD: Mean difference
OR: Odds ratio
PRISMA: Preferred Reporting in Systematic Reviews and Meta analyses
RCT: Randomised controlled trial
RR: Risk ratio
SD: Standard deviation
SMD: Standardised mean difference
TBA: Traditional Birth Attendant
