# Supplementary material for: Systematic review of community participation interventions to improve maternal health outcomes in rural South Asia
Source: BMC Pregnancy Childbirth. 2018 Aug 10;18:327. doi: 10.1186/s12884-018-1964-1 (PMC6086057; doi:10.1186/s12884-018-1964-1)
Supplement: Supplementary file 1 — The Medline search strategy. (DOCX 19 kb) [file 12884_2018_1964_MOESM1_ESM.docx]

The Medline search strategy

A three-step search strategy was used. Firstly, an initial search of Medline (OVID 1946+) was undertaken, followed by analysis of words included in the title and abstract, and MESH headings used to index relevant articles. The Medline search was modified to include additional relevant keywords and MESH headings.

The Medline search was then adapted using both keyword and subject headings to the following health-specific and multidisciplinary databases:

- CINAHL (Ebsco 1937+)
- Cochrane Library (Wiley)
- Embase (OVID 1947+)
- Maternity and Infant Care Database (OVID 1971+)
- Medline in Process (OVID 1946+)
- POPLINE (K4Health 1827+)
- Scopus (Elsevier 1823+)
- Western Pacific Region Index Medicus (WHO)

Database searches were initially conducted in November 2015, and updated in June 2017. No restriction was placed on language or year of publication.

The search strategy included the use of terms in four broad domains:

1. Family and community: including community networks, community health services, rural health services
2. Maternal health care: including prenatal and perinatal care, pregnancy, delivery and maternal health services

(3) Maternal mortality: including pregnancy complications, pregnancy outcome, maternal death

(4) South East Asian countries: Nepal, Pakistan, Bhutan, India, and Bangladesh.

Hand searches were conducted on the reference lists of included studies.

Database(s): MEDLINE 1946 to Present

| **No** | **Searches** |
| --- | --- |
| 1 | Community Health Services/ |
| 2 | Community Networks/ |
| 3 | Consumer Participation/ |
| 4 | Patient Participation/ |
| 5 | Community Health Planning/ |
| 6 | ((communit* or village* or famil* or local people* or resident* or public or home* or household* or husband* or male* or spouse* or partner* or rural or remote* or marginali?ed or distance*) adj5 (educat* or strateg* or intervention* or prevent* or program* or campaign* or support* or manage* or healthcare* or health or involve* or aware* or participat*)).tw. |
| 7 | Rural Health Services/ |
| 8 | health education/ or patient education as topic/ or prenatal education/ |
| 9 | 1 or 2 or 3 or 4 or 5 or 6 or 7 or 8 |
| 10 | Maternal Welfare/ |
| 11 | Prenatal Care/ |
| 12 | Perinatal Care/ |
| 13 | Maternal Health Services/ |
| 14 | Pregnancy/ |
| 15 | Delivery, Obstetric/ |
| 16 | (prenatal or perinatal or antenatal or birth* or deliver* or pregnan*).tw. |
| 17 | ((traditional or skilled) adj5 (attendant* or assistant*)).tw. |
| 18 | ((mother* or matern*) adj5 (health* or welfare or care*)).tw. |
| 19 | Maternal-Child Health Centers/ |
| 20 | 10 or 11 or 12 or 13 or 14 or 15 or 16 or 17 or 18 or 19 |
| 21 | Maternal Mortality/ |
| 22 | Pregnancy Complications/ |
| 23 | Pregnancy Outcome/ |
| 24 | Maternal Death/ |
| 25 | ((mother* or matern* or pregnan* or reproduct* or birth*) adj5 (death* or mortalit* or fatal* or outcome* or complicat*)).tw. |
| 26 | 21 or 22 or 23 or 24 or 25 |
| 27 | 20 or 26 |
| 28 | randomized controlled trial.pt. |
| 29 | controlled clinical trial.pt. |
| 30 | random*.tw. |
| 31 | trial.tw. |
| 32 | groups.tw. |
| 33 | ((them* or content) adj5 analy*).tw. |
| 34 | Qualitative Research/ |
| 35 | qual*.tw. |
| 36 | ((observation* or comparative or multicenter or multicentre) adj5 (research or method* or stud*)).tw. |
| 37 | (participant* adj5 observ*).tw. |
| 38 | (field adj5 (stud* or note* or research or method*)).tw. |
| 39 | theme*.tw. |
| 40 | observation*.tw. |
| 41 | 28 or 29 or 30 or 31 or 32 or 33 or 34 or 35 or 36 or 37 or 38 or 39 or 40 |
| 42 | Nepal/ |
| 43 | Pakistan/ |
| 44 | Bhutan/ |
| 45 | India/ |
| 46 | Bangladesh/ |
| 47 | (nepal* or pakistan* or bhutan* or india* or banglad*).tw. |
| 48 | 42 or 43 or 44 or 45 or 46 or 47 |
| **49** | **9 and 27 and 41 and 48** |
