# Supplementary material for: Systematic review of community participation interventions to improve maternal health outcomes in rural South Asia
Source: BMC Pregnancy Childbirth. 2018 Aug 10;18:327. doi: 10.1186/s12884-018-1964-1 (PMC6086057; doi:10.1186/s12884-018-1964-1)
Supplement: Supplementary file 2 — Title of data: GRADE tables. List of outcomes, relative effects (95% CI), number of participants (studies) and certainty of the evidence (GRADE). (PDF 73 kb) [file 12884_2018_1964_MOESM2_ESM.pdf]

Outcomes	Relative effect (95% CI)	№ of participants (studies)	Certainty of the evidence (GRADE)
Antenatal Care (any) adjusted RR - Community mobilisation	RR 1.28 (1.13 to 1.46)	9644 (2 RCTs)	⨁⨁⨁◯ MODERATE [a]
Antenatal Care (any) adjusted RR - Community mobilisation home care using male and female community mobilisers	RR 1.47 (1.21 to 1.78)	3449 (1 RCT)	⨁⨁⨁◯ MODERATE [a]
Antenatal Care (any) adjusted RR - Community mobilisation community care using female community mobilisers	RR 1.13 (0.93 to 1.36)	3350 (1 RCT)	⨁⨁⨁◯ MODERATE [a]
Antenatal Care (any) adjusted RR - Community education	RR 1.40 (1.12 to 1.75)	3491 (1 RCT)	⨁⨁⨁⨁ HIGH
Antenatal Care (any) adjusted RR - Women's education groups	RR 1.06 (0.91 to 1.23)	61896 (5 RCTs)	⨁⨁⨁◯ MODERATE [a]
Antenatal Care (any) adjusted RR - Women's and men's education groups	RR 1.35 (0.81 to 2.25)	1728 (1 RCT)	⨁⨁◯◯ LOW [a,b]
Antenatal Care (≥ 3 visits) - All community interventions	RR 1.01 (0.78 to 1.31)	60307 (5 RCTs)	⨁⨁⨁◯ MODERATE [a]
Antenatal Care (≥ 3 visits) - Community mobilisation	RR 1.44 (0.75 to 2.77)	4534 (1 RCT)	⨁⨁⨁◯ MODERATE [b]
Antenatal Care (≥ 3 visits) - Women's education groups	RR 0.74 (0.54 to 1.02)	55773 (4 RCTs)	⨁⨁⨁◯ MODERATE [a]
Skilled birth attendant - Skilled birth attendant (any)	RR 1.24 (0.39 to 3.90)	24047 (2 RCTs)	⨁◯◯◯ VERY LOW [a,b,c]
Skilled birth attendant - Skilled birth attendant (formal provider)	RR 1.02 (0.70 to 1.48)	72347 (4 RCTs)	⨁⨁⨁⨁ HIGH
Skilled birth attendant - Skilled birth attendant (traditional birth attendant)	RR 1.14 (0.60 to 2.17)	21333 (2 RCTs)	⨁⨁⨁◯ MODERATE [a]
Delivery at a health facility (all adjusted RR) - Women's education groups	RR 1.15 (1.11 to 1.20)	36989 (2 RCTs)	⨁⨁⨁◯ MODERATE [a]
Maternal deaths (N = number of live births) STATA one way ICC - Maternal deaths over 2 years	RR 0.63 (0.24 to 1.64)	61487 (5 RCTs)	⨁◯◯◯ VERY LOW [a,c]
Maternal deaths (N = number of live births) STATA one way ICC - Maternal deaths over 3 years	RR 1.11 (0.52 to 2.36)	48921 (2 RCTs)	⨁⨁⨁◯ MODERATE [a]

Explanations

a. risk of bias due to incomplete follow-up

b. imprecise estimate

c. effect estimates of individual studies in the meta-analysis vary between benefit and harm
